# Redox potential research in the field of balneochemistry: case study on equilibrium approach to bioactive elements in therapeutic waters

**DOI:** 10.1007/s00484-020-01871-7

**Published:** 2020-02-11

**Authors:** Katarzyna Wątor, Dariusz Dobrzyński, Kenji Sugimori, Ewa Kmiecik

**Affiliations:** 1 Faculty of Geology, Geophysics and Environmental Protection, AGH — University of Science and Technology, Mickiewicza 30 Av., 30-059 Kraków, Poland; 2 Faculty of Geology, University of Warsaw, Warsaw, Poland; 3 Department of Biology, Faculty of Medicine, Toho University, Tokyo, Japan

**Keywords:** Groundwater geochemistry, Therapeutic water, Oxidation-reduction potential, Geochemical modelling, Iron, Sulphur

## Abstract

In some countries (e.g. Poland, Czechia, Slovakia, Russia, Germany), oxidation-reduction potential (ORP) measurements are required to document the quality of groundwater which are planned to be used as therapeutic waters. ORP is still rarely studied and not fully availed in therapeutic water research. Studies of ORP in various types of therapeutic, mineral and thermal waters in sites of Poland integrated with geochemical equilibrium approach were employed to characterize two redox-sensitive and bioactive elements, i.e. iron and sulphur. Studied waters present reducing conditions (*E*_H_ between − 406 and − 41 mV) at outflow or extraction sites; however, they significantly differ in terms of total dissolved solids, temperature, and iron, sulphur(II) and sulphate concentrations. These result in recognizable differences, e.g. in terms of saturation state with respect to aquifer rock minerals and the dominating forms of occurrence of elements studied disclosed on the stability field diagrams. Considering the methodological determinants, ORP orchestrated with geochemical modelling tools might be successfully applied for studying natural linkages between various groundwater in natural systems, protecting the therapeutic water resource, and identifying the changes of water quality both at exploitation sites (springs, wells) and treatment places.

## Introduction

The oxidation-reduction potential (ORP, redox), a parameter in the canon of physicochemical tests in groundwater, is also very important because releasing of many elements of health concern (e.g. As, Cr, N, Se, U) from aquifer rocks into groundwater is a redox-dependent process (Jacks 2017). Furthermore, legal regulations, as e.g. in Poland (DMH 2006), Czechia (Vyhlaska 2001), Slovakia (Vyhlaska 2006), Russia (Classification 2000), Germany (Begriffsbestimmungen 2016), require ORP testing as one of the parameters for documenting the physicochemical characteristics of groundwater which are planned to be used as therapeutic (medicinal, curative) ones. However, ORP is rarely studied in therapeutic waters and the archived outcomes are seldom disseminated in publications. This allows the opinion that ORP studies in therapeutic waters are still poorly benefited from in the field of therapeutic water-related research (Dobrzyński et al. 2018).

The chemical composition of groundwater in both pristine and contaminated aquifer systems is affected by oxidation-reduction (redox) processes. Redox reactions have a controlling influence on the solubility and transport of species of numerous major and minor chemical elements in natural waters. Aqueous species of elements can be quantified by chemical speciation analysis or estimated by speciation modelling based on chemical thermodynamics. The geochemical speciation modelling is a tool that, among others, allows estimating species activities and the saturation state of waters with respect to solid phases. Such calculations for electro-active elements require reliable information about the ORP of a solution (Langmuir 1997; Drever 1997; Appelo and Postma 2005; Merkel and Planer-Friedrich 2008).

Among the components that give therapeutic properties to groundwater, iron and compounds of sulphur(II) are commonly considered (e.g., DMH 2006; GMA 2019). For instance, in Poland, the established threshold values for ferrous and sulphurous therapeutic waters are 10 mg L^−1^ of Fe^2+^ and 1 mg L^−1^ of S(II) compounds, respectively. Increased concentrations of S(II) commonly coexist with high concentrations of other sulphur solutes, mostly sulphates. Iron, sulphur(II) and sulphates are therapeutically active components with very well-documented benefits in balneotherapy (e.g. Gutenbrunner and Hildebrandt 1994; Costantino et al. 2006; Zámbó et al. 2008). Both iron and sulphur are electro-active (redox-sensitive) chemical elements common in hydrogeochemical systems, such as therapeutic, mineral or thermal water aquifers.

One of the basic requirements which therapeutic waters have to meet is the stability of their chemical composition, understood as fluctuations within the limits of natural changes. This assessment requires regular monitoring of physical and chemical parameters. The ORP parameter sensitive to external conditions can be used as an indicator of the therapeutic water contamination and/or the quality changes induced by the regime of water exploitation. Variations of ORP in the aquifer or the near-well environment might affect the distribution of iron and sulphur species in exploited therapeutic water. Adverse features of the chemical composition of therapeutic waters caused by changes in redox conditions might also happen while handling water between the exploitation sites and the treatment places (Okouchi et al. 1998; Dobrzyński et al. 2013).

The issues indicated above should be considered in the balneochemical assessment of natural therapeutic raw materials, when by balneochemistry is meant a field of balneology focused e.g. on the chemical composition and both physical and chemical features of therapeutic materials as waters, gases and peloids (Ponikowska 2015).

The paper aims (1) speciation-solubility characterizing of redox-sensitive bioactive elements (iron and sulphur) in selected therapeutic waters by the tools of geochemical modelling, (2) evaluating the usefulness of equilibrium approach for documenting and monitoring the quality of groundwater used as therapeutic water, and also (3) paying the wider attention to the need and the importance for the redox research in therapeutic waters. For these purposes, the results of authors’ studies on various types of groundwater (therapeutic, mineral and thermal) from sites selected in Poland were used.

## Essentials of oxidation-reduction potential in groundwater

The ORP investigations are an important part of groundwater geochemistry. Two approaches for characterizing redox processes in geochemical systems were developed: an equilibrium approach and a kinetic one (Chapelle 2004). The equilibrium approach is based on methods of physical chemistry. To describe redox processes, theoretical activity of electrons in aqueous solution (pe) is used as a master variable, which is a function of redox potential difference (*E*_H_):

1 $$ \mathrm{pe}=\frac{F}{2.303 RT}{E}_{\mathrm{H}} $$

where pe is the negative logarithm of electron activity (1), *E*_H_ is the redox potential difference (V) measured and next corrected with respect to the standard hydrogen electrode, *F* is the Faraday constant 9.64853 · 10^4^ C/mol, *R* is the gas constant 8.31447 J/(mol · K) and *T* is the absolute temperature (K).

The *E*_H_ of the solution is related to concentrations of aqueous redox couples (e.g. ${\mathrm{NO}}_3^{-}/{\mathrm{NH}}_4^{+}$; Fe^3+^/Fe^2+^; Mn^4+^/Mn^2+^; ${\mathrm{SO}}_4^{2-}/{\mathrm{H}}_2\mathrm{S}$) at chemical equilibrium and voltage of a standard hydrogen electrode according to the Nernst equation (e.g. Appelo and Postma 2005).

This parameter is significantly related to the pH because in many reactions simultaneous transfer of both electrons and protons occurs, and the relationship between *E*_H_ and pH depends on the ratio of protons to electrons transferred.

The *E*_H_ is defined as a unique value only when a system is at thermodynamic equilibrium. If the activities of ions in the redox couple are not at equilibrium, an infinite number of *E*_H_ values can be measured or calculated, but will not be the same *E*_H_ as defined by the Nernst equation. It could be possible to define the *E*_H_ of the solution as a whole only when *E*_H_ values determined by the equilibrium of each couple were the same. However, natural water systems are often not at redox equilibrium and the various redox couples gave widely different *E*_H_ values (Lindberg and Runnells 1984). Numerous studies have also shown that *E*_H_ measurement with platinum electrodes was not consistent with *E*_H_ calculated from the Nernst equation.

Various factors affect the quality of ORP measurements. For example, the effect of so-called electrode poisoning often occurs in sulphurous water and can cause more negative potentials (Whitfield 1974). Inserting (un-rinsed off) redox electrode into water rich in iron directly after measurement in ZoBell solution causes the formation of coatings and erratic results (Nordstrom and Wilde 2005). The studies on the ORP of hot springs in Taiwan and Iceland prove that some sensors used for *E*_H_ measurements are very sensitive to HS^−^/SO_4_^2−^ redox couple (Chen and Sung 2009) and are not responded to selected other redox couples (Stefánsson et al. 2005). Redox reaction of iron sulphide/sulphate ions is dominant even if concentrations of sulphides and sulphates are below the detection limits of the analytical methods (Hokari et al. 2014). The important role of iron and sulphide in *E*_H_ determination was also emphasized (e.g. Grenthe et al. 1992; Ioka et al. 2011, 2017). The other factor affecting ORP measurements is the time of result stabilization. Tests conducted by Gómez et al. (2006) and Ioka et al. (2011, 2016) indicate that sometimes the stable values might be observed after at least a week.

The above issues arise mainly from the following reasons:

1. Groundwater usually contains multiple redox species couples that are not in conjoint equilibrium (e.g. Lindberg and Runnells 1984). One of the reasons is that the achieving of redox equilibrium is a slow process. Many studies prove that redox species can coexist at disequilibrium, e.g. dissolved oxygen coexists with hydrogen sulphide, methane and ferrous iron. Consequently, a single value of *E*_H_ cannot characterize a disequilibrium system.
2. The *E*_H_ measuring with the platinum electrode relates to analytical difficulties (e.g. Appelo and Postma 2005). The redox electrode responds to electron transfers between solutes (Thorstenson 1984). However, the Pt-electrode responds satisfactorily to a few of the redox couples important in natural waters (Drever 1997). A platinum electrode readily responds to concentrations of ions that react rapidly and reversibly with platinum (like Fe^2+^ and Fe^3+^). The Pt-electrode is relatively insensitive to the O_2_/H_2_O and CO_2_/CH_4_ redox couples because solutes such as oxygen, carbon dioxide and methane react sluggishly on a platinum surface. Hence, redox potentials measured are generally lower than real values, even when equilibrium is reached.
3. Many of the redox processes occurring in groundwater systems are driven by microbial activity (e.g. Lovley et al. 1989; Blöthe and Roden 2009). Using the *E*_H_ value to describe redox processes catalyzed by microorganisms violates the assumption of redox equilibrium (e.g. Chapelle 2001, 2004). However, microorganisms can actively respire and reproduce only when there is available free energy to drive their metabolism. That is, microorganisms require that their environment is not at thermodynamic equilibrium.

In this work, the equilibrium approach was applied for the interpretation of sulphur and iron species in studied groundwater.

The equilibrium approach is not the only way to describe redox processes in groundwater systems. The metabolism of microorganisms is based on the cycling of electrons from electron donors (often organic carbon) to electron acceptors such as molecular oxygen, nitrate, ferric iron, sulphate and carbon dioxide. Microorganisms capture this electrical energy related to the flow of electrons, convert it to chemical energy, and use it to support life functions. If it is assumed that redox processes in groundwater systems are driven predominantly by microbial metabolism, it becomes possible to describe these processes by the cycling of electron donors, electron acceptors and intermediate products of microbial metabolism (Lovley et al. 1994; Chapelle 2001, 2004). Because this is a non-equilibrium, kinetic description, it is termed the “kinetic approach”.

## Materials and methods

The groundwater of various compositions and ages derived from reducing hydrogeochemical environments were selected for analysis. In general, these are therapeutic waters. Some of them are thermal waters used for recreational purposes. In total, fifty-one physicochemical analyses of waters from intakes located in two areas, the Sudetes Mountains (12 analyses) and the Carpathian Foredeep (39 analyses), were interpreted (Table 1).

**Table 1:** Selected physicochemical characteristics of studied therapeutic, mineral and thermal waters

Region	Total dissolved solids (g L^−1^)	pH	*E*_H_ (mV)	*T* (°C)	SO_4_ (mM)	S(II) (μM)	Fe (μM)	Hydrochemical types^1^
Sudetes Mountains–Jelenia Góra Valley (*N* = 9)	0.4 to 0.6	7.96 to 8.66	− 165 to − 59	48.2 to 79.4	0.85 to 1.88	0.62 to 4.68	< 0.18 to 0.54	SO_4_-HCO_3_-Na, F, Si and HCO_3_-SO_4_-Na
Sudetes Mountains–Kłodzko region (*N* = 3)	0.2	9.19 to 9.22	− 160 to − 124	43.0 to 44.3	0.11 to 0.16	45.23 to 114.2	< 0.18 to 0.66	HCO_3_-F-Na, S, Si, Rn
Carpathian Foredeep–Busko region (*N* = 29)	12.1 to 34.4	6.51 to 7.60	− 406 to −100	10.8 to 23.8	14.7 to 40.6	913 to 30,000	< 0.2 to 17.9	Cl-Na, S, I
Carpathian Foredeep–Kraków region (*N* = 10)	1.7 to 2.7	6.70 to 7.59	− 179 to − 41	10.0 to 12.9	5.4 to 15.8	99 to 2580	0.4 to 8.2	SO_4_-Cl-Na-Mg-Ca, S; SO_4_-HCO_3_-Ca-Mg, S and SO_4_-Ca-Mg, S

^1^Name of hydrochemical types contain main solutes (of more than 20% equivalents of anions and cations, respectively) and bioactive specific components above threshold values (after DMH 2006), i.e. 1 mg/L of S(II) compounds, 2 mg L^−1^ of F, 70 mg/L of Si (as H_2_SiO_3_), and 1 mg L^−1^ of I, respectively

Thermal fresh waters of the Sudetes Mountains (SM) occur in crystalline aquifer rocks. In Jelenia Góra Valley, thermal waters of likely last glacial age occur in Carboniferous granitoides. Thermal waters of Lądek-Zdrój (the eastern part of the Kłodzko region) which have presumably early Holocene age occur in Palaeozoic gneisses. Studied mineral waters of the Carpathian Foredeep (CF) occur in sedimentary rocks of different ages. In the area of Busko-Zdrój, mineral waters of before-Holocene, presumably last glacial age, occur in Jurassic and Cretaceous aquifer rocks. Mineral waters of the Kraków region of glacial and early Holocene ages, locally with a small component of modern water, are generally related to Miocene aquifer rocks.

Mineral waters from the CF are characterized by the mineralization (total dissolved solids) between 1.72 and 34.4 g L^−1^, temperature from 10.0 to 23.8 °C, pH in the range 6.51–7.60 and ORP between − 406 and − 41 mV. The concentrations of sulphur(II) compounds, sulphates and iron in these waters change from 0.1 to 30 mM, from 5.4 to 40.6 mM and from below 0.2 to 17.9 mM, respectively. The SM thermal waters have mineralization between 0.2 and 0.6 g L^−1^, temperature from 43.0 to 79.4 °C, pH between 7.96 and 9.22 and ORP in the range from − 165 to − 59 mV. Concentrations of sulphur(II), sulphates and iron in the SM waters vary from 0.6 to 114 μM, from 0.1 to 1.9 mM and from below 0.18 to 0.66 μM, respectively. Due to significant differences in the chemical composition of waters from both areas, the interpretation for them was carried out separately.

The measurements of *E*_H_ were carried out without atmosphere contact in a through-flow chamber with using platinum Ag/AgCl electrode and corrected concerning to standard hydrogen electrode. Numerous factors affect *E*_H_ measurement and therefore influence the outcomes of geochemical modelling. In the case of the occurrence of sulphides in water, it is very easy to make erroneous measurement due to electrode poisoning (Whitfield 1974; Peiffer et al. 1992). Determination of the sulphur(II) compounds, sulphates and iron can be biased by the errors made during sample collection, sample preparation and analysis (Kmiecik 2018). Thus, the appropriate and standardized methods of sample collection and analysis should be applied. All samples were collected in accordance with the requirements described in ISO 5667-11 standard. Samples for sulphates and iron determination were filtrated in the field with the use of MF-Millipore MCE membrane filters with a 0.45-μm pore size and acidified with concentrated nitric acid (J.T. Baker). Samples for sulphide analysis were collected to the dark glass bottle without the air and preserved by the addition of disodium EDTA. The analyses of sulphates and iron in water samples from the Carpathian Foredeep were performed using inductively coupled plasma optical emission spectrometry (ICP-OES) according to 11885 ISO standard. The Optima 7300 DV (Perkin Elmer, USA) spectrometer was used. The analytical emission spectrum line of 181.975 nm was applied for sulphur (recalculated to sulphate ions) and 238.204 nm for iron indications. Quantization was achieved by 5-point external calibration curve basing on 4 standard solutions and blank sample analysis. Multi-elemental and one-element standard solutions were used from the Merck company (Germany). Deionized ultrapure water (18.2 MΩcm) was obtained with the Milli-Q system (Millipore, MA). Concentrations of sulphur(II) compounds were determined with the thiomercurimetric titration method. Sulphide IC standard from Sigma-Aldrich was used as a control sample. In waters from the Sudetes, analyses of iron were performed by inductively coupled plasma mass spectrometry (in Acme Labs, Canada), and sulphates and sulphur(II) compounds determined by using DR-3000 spectrophotometer (HACH, USA).

Chemical analyses of groundwater were used for geochemical speciation-solubility modelling performed by programmes: the PHREEQC, v.3.0 (Parkhurst and Appelo 2013) with *llnl.dat* thermodynamic database, and by the Geochemist’s Workbench, ver. 12 (Bethke et al. 2018) with *thermo.dat* database. Geochemical modelling calculations are based on the assumption of chemical equilibrium between solutes in solution.

The *E*_H_ and pH measurement results were plotted on stability field *E*_H_-pH diagrams constructed by using the Geochemist’s Workbench program. Stability field diagrams were created for mean molar concentrations of S(II), S(VI) and Fe(II), and at mean temperatures, in both groups of waters, i.e. thermal fresh waters from the Sudetes Mountains (for mean temperature about 50 °C) and mineral sulphate-rich waters from the Carpathian Foredeep Basin (average 13 °C). The *E*_H_-pH diagrams indicate (1) predominant aqueous species of the analyzed chemical element(s) and (2) most stable (supersaturated) solids limiting/affecting concentrations of the element(s).

## Results and discussion

**Fig. 1:**
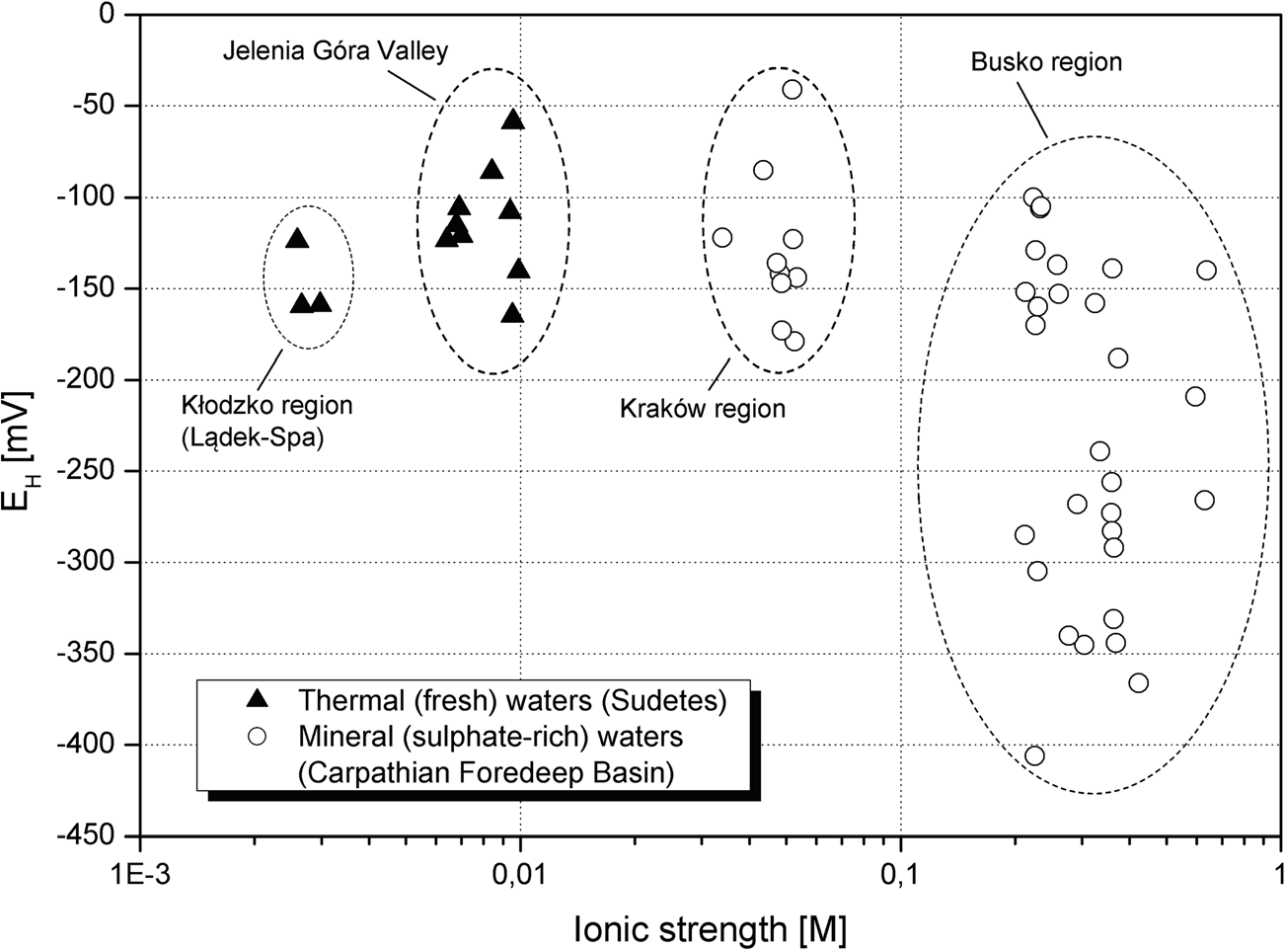
Oxidation-reduction potential vs. ionic strength of studied groundwater

**Fig. 2:**
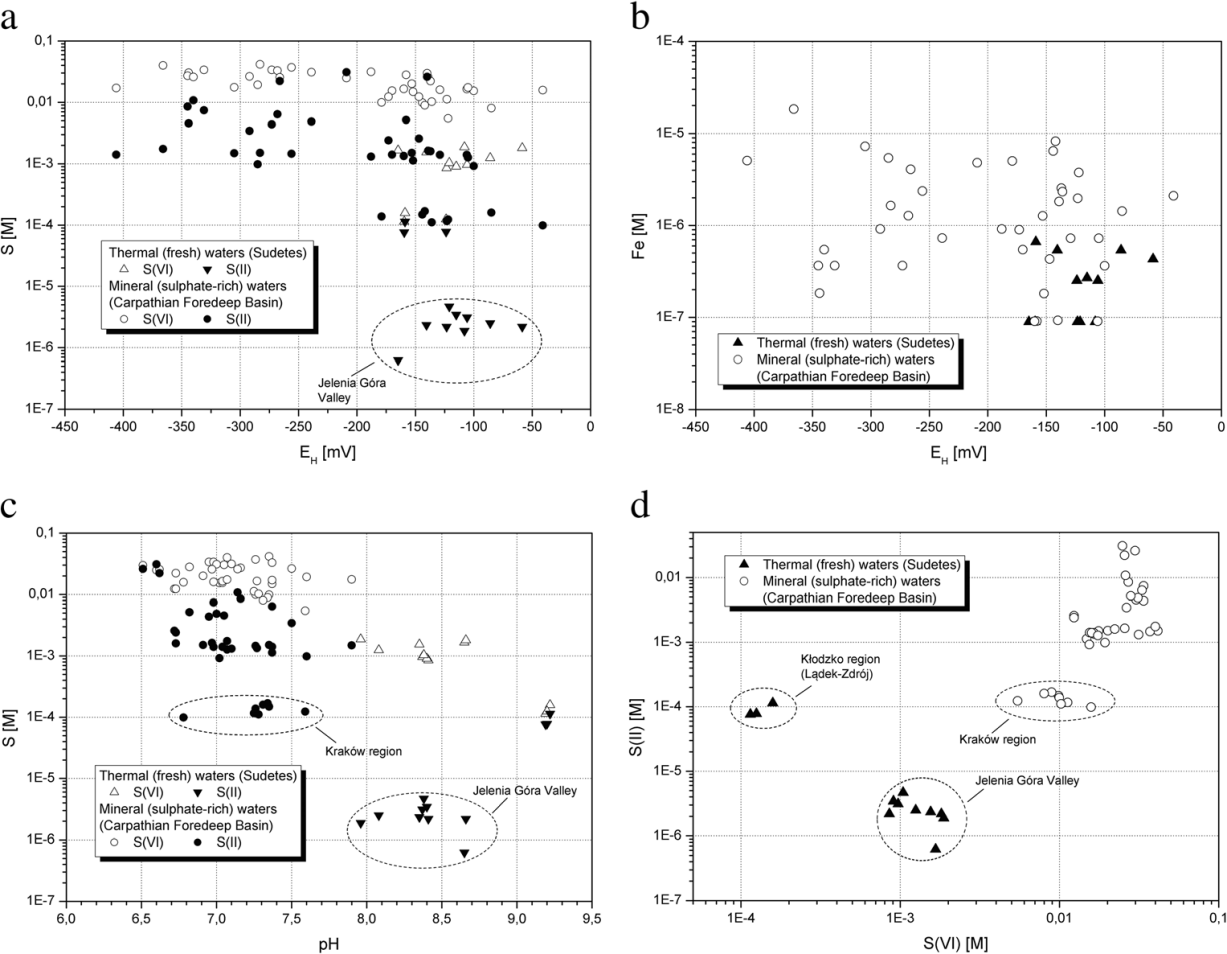
Relations between selected physicochemical characteristics in groundwater: **a** sulphur vs. oxidation-reduction potential, **b** iron vs. oxidation-reduction potential, **c** sulphur vs. pH, and **d** sulphur(II) vs. sulphur(VI)

Thermal waters from the Sudetes Mountains (SM) are characterized by lower ionic strength and higher ORP value than most of the waters from the Carpathian Foredeep (CF) Basin (Table 1, Fig. 1). In the analyzed group of mineral waters from the CF, greater ORP dispersion is observed with higher values of ionic strength. The variation of ORP values in CF waters affects the concentration of S(II) and S(VI) compounds (Fig. 2a). With the increase of ORP, the total amount of sulphates and S(II) compounds decreases. Similar dependence can be seen in the case of total iron concentration (Fig. 2b). The absolute difference between concentrations of S(VI) and S(II) decreases with the pH lowering (Fig. 2c). In mineral waters from the CF Basin, the growth of S(II) compounds is also observed while higher concentrations of sulphates occur (Fig. 2d). There is no clear dependency between iron content and sulphur concentrations. The oxidation process is based on increasing concentrations of S(VI) and decreasing the values of Fe(II). However, the research based on the comparison of the *E*_H_ values measured in the field and estimated in PHREEQC programme with the use of concentration Fe^2+^/Fe^3+^ redox couple shows a more reduced state for calculated *E*_H_ (Hokari et al. 2014) and might suggest lack of equilibrium between Fe^2+^/Fe^3+^ and SO_4_^2−^/HS^−^ redox pairs.

The total effects of chemical interactions in the groundwater-aquifer rock systems can be quantified by saturation index (SI) which shows the relation between real saturation state with respect to the solid and hypothetical equilibrium state:

2 $$ \mathrm{SI}=\log \frac{\mathrm{IAP}}{K_{\mathrm{T}}} $$

where IAP is the ion activity products (the product of the activities of forms involved in the reaction) and *K*_T_ is the equilibrium constant of reaction at the given temperature.

**Fig. 3:**
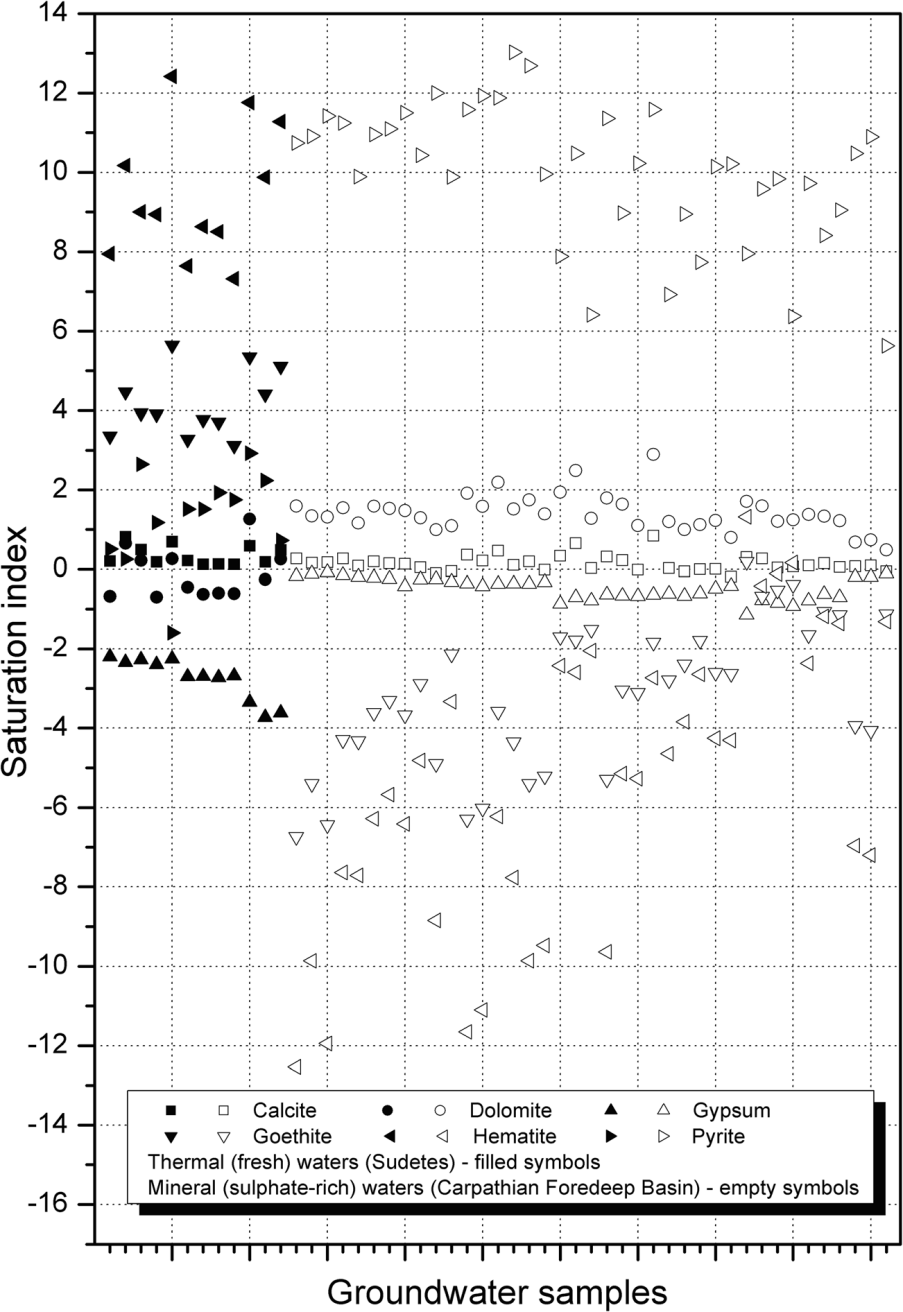
Saturation indices of studied groundwater with respect to selected minerals

The most common and/or reactive minerals forming studied aquifer rocks were selected for this assessment. The studied groundwater represents various geological settings and aquifer rocks. The common feature of studied waters is chemical equilibrium in relation to calcite (Fig. 3) despite significantly various pH and aqueous chemistries. In sedimentary aquifer rocks of the CF Basin, calcite is a common rock-forming mineral. In crystalline aquifer rocks of thermal waters in the SM, calcite is a very rare secondary mineral. Howbeit, thermal fresh waters are also equilibrated with calcite because of alkaline pH (8.0–9.2). Richer in magnesium, mineral waters of CF (53–1113 mg L^−1^) are oversaturated with respect to dolomite, whereas SM waters are very poor in this element (from below 0.05 to 0.24 mg L^−1^ of Mg) and remain undersaturated (Fig. 3).

All analyzed waters are supersaturated with respect to pyrite, whereas CF waters show significantly higher oversaturation due to much higher S(II) concentrations than in SM thermal waters. Mineral waters from the CF are only slightly undersaturated with gypsum, which is a common mineral in these sedimentary aquifers. Gypsum is a very uncommon mineral in SM crystalline aquifer rocks what is likely the main reason for groundwater undersaturation.

Significant differences of pH and S(II) concentrations between both water groups respond for extremely different saturation state with respect to common iron-bearing solids (goethite, haematite) (Fig. 3), despite that, all studied waters are poor in iron. The alkaline and S(II)-poor SM thermal waters are oversaturated with goethite and haematite, whereas near-neutral and S(II)-very rich CF mineral waters are highly undersaturated with both minerals.

Stability field diagrams indicate the predominant (most abundant) aqueous species of chemical element and the solid phases (solid species) with respect to the waters are most saturated (i.e. most stable solids). The *E*_H_-pH diagrams have conditionings and limitations. Diagrams are drawn to show equilibrium relations (conditions), but equilibrium is often not attained in aquifer systems, especially of the short turn-over time zone. In studied groundwater, chemical equilibria could be expected because of water old ages, the deepwater circulation systems and/or the presence of reactive minerals in the aquifer rocks. Field boundaries in diagrams are calculated for equilibrium conditions with the assumption that reacting species occur at equal concentrations which is usually not met in natural systems. Diagrams do not take account of slow reactions or metastable forms of chemical elements. Next, uncertainty of thermodynamic data and range and incompatibility of databases also pose restraints of diagrams. However, apprehending conditionings, such diagrams are useful when the occurrence of different forms of selected elements in water analyzed is considered (e.g. Gómez et al. 2006; Hokari et al. 2014; Ioka et al. 2017). Changes of water quality in terms of *E*_H_-pH relations can be easily recognized by using such diagrams in monitoring adverse effects, e.g. in therapeutic waters affected by chlorination (Okouchi et al. 2005).

**Fig. 4:**
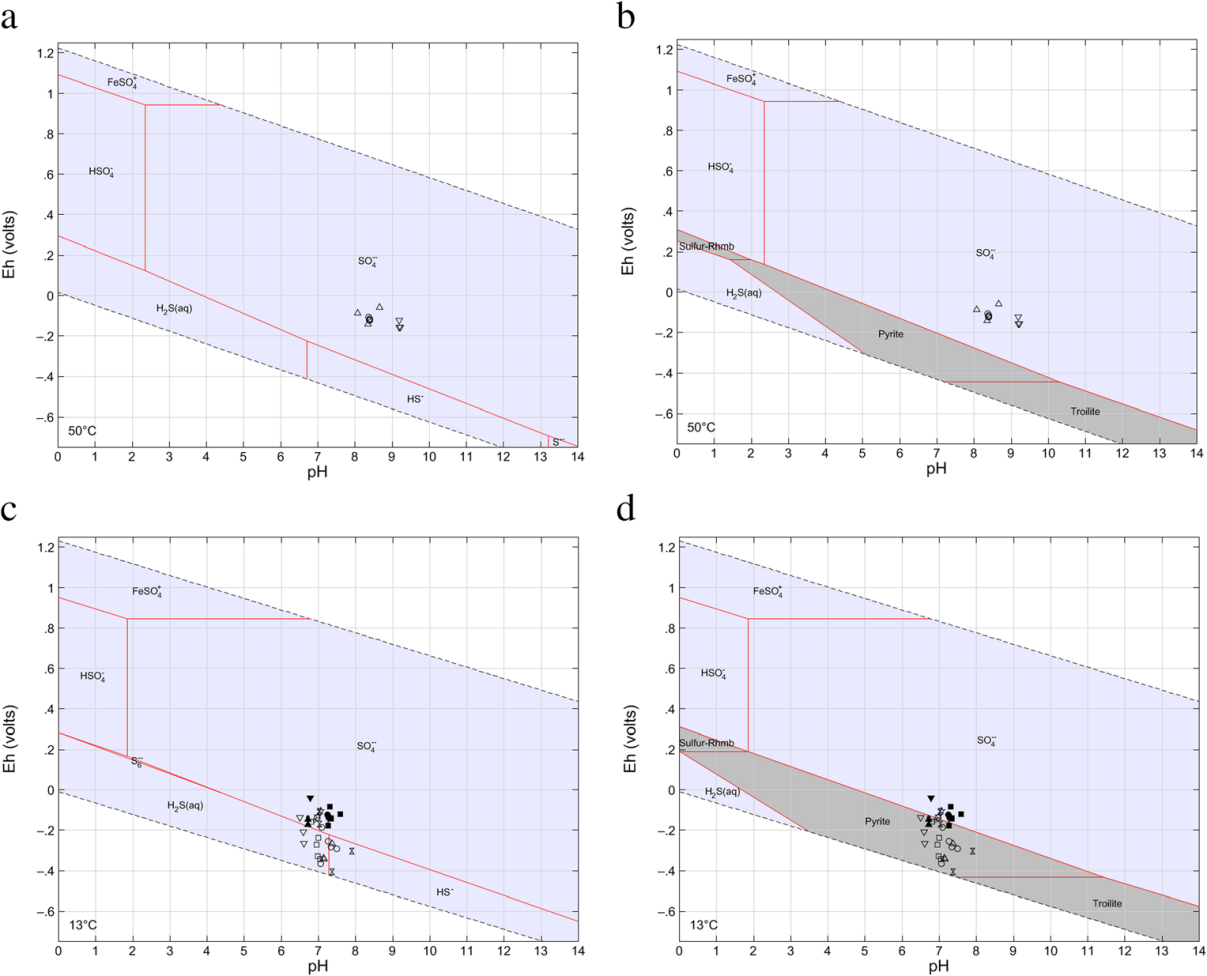
*E*_H_-pH diagrams of the sulphur species in groundwater at the S-Fe-O-H system. Thermal fresh waters (Sudetes Mountains), without water from C-1 intake (in Cieplice), at 1 mM S(VI), 0.3 μM Fe, 50 °C and 1 bar total pressure: **a** aqueous species and **b** aqueous and solid species. Mineral waters (Carpathian Foredeep), at 20 mM S(VI), 2 μM Fe, 13 °C and 1 bar total pressure: **c** aqueous species and **d** aqueous and solid species. Filled symbols, waters of Kraków region

Predominant sulphur aqueous species in the SM waters is SO_4_^2−^ (Fig. 4a, b). Both diagrams were constructed for the temperature of 50 °C (average for most of the SM waters). Water from C-1 well (Cieplice) which has a much higher temperature (of around 78–79 °C at the outflow) is not shown in Fig. 4a and b. Nonetheless, the water of C-1 well presents the same feature as other SM thermal waters. CF mineral waters generally present conditions of equilibrium between H_2_S_(aq)_, HS^−^ and SO_4_^2−^ species. Waters of Kraków region (occurring in Miocene aquifer rocks) which have lower S(VI) concentrations indicate SO_4_^2−^ as the predominant species (Fig. 4c). Chemistry of most of the CF waters shows pyrite as the most stable sulphur-solid (Fig. 4d). The occurrence of H_2_S plays an essential role in the beneficial effects of sulphurous mineral waters. This form is more available in the environment with a low pH and low oxygen concentrations (Carbajo and Maraver 2017). Even that the impact of pH on the S(II) species is usually discussed in literature due to their behaviour in different parts of the human body (op. cit.), the role of ORP should also be considered when such therapeutic waters are used.

ORP informs about physicochemical properties of water used for treatments and influences the aqueous speciation of chemical elements, what consequently might also affect therapeutic effectiveness. For instance, Takahashi et al. (2007) documented the influence of bathing in waters of different ORP and ionic compositions on the renal system, uric acid excretion and urine pH, and also on the electrical potential of the epidermal cell membranes. It is important to remember that the ORP of water changes during water handling and ageing. Fresh water samples of thermal and therapeutic water have usually low ORP value and its growth during water storage. This results in changes of S(II) and Fe species distribution and, as a consequence, in curative properties of water (Okouchi et al. 2010). That is why the ORP measurements should be performed as well on water wellheads as in the places where treatments are carried out (Ohnami et al. 2008b). ORP research at treatment sites confirmed also usability in the case of assessing the scale of adverse physicochemical changes in thermal water chemically modified by the addition of an artificial additive (Ohnami et al. 2008a).

Sulphur compounds (especially hydrogen sulphide—H_2_S) in therapeutic/thermal waters show a very well-documented beneficial therapeutic effect (e.g. Nasermoaddeli and Kagamimori 2005; Legwant et al. 2013; Vela-Anero et al. 2017; Gálvez et al. 2018). However, hydrogen sulphide might also create a potential risk for water users. Sporadic tragic incidents (Bassindale and Hosking 2011; Stanhope et al. 2017) show that it is necessary to monitor the hydrogen sulphide in waters and indoor atmosphere in places where H_2_S-rich waters are used for therapeutic or recreational purposes. One allergic reaction to sulphur was observed during studies conducted by Bender et al. (2014). Varga (2012) proposed a complex strategy for balneoprevention including calculation of toxicological risk of balneological treatment based on a complete chemical analysis (including organics) and specific toxicity test to avoid the negative consequences of the use of therapeutic waters.

**Fig. 5:**
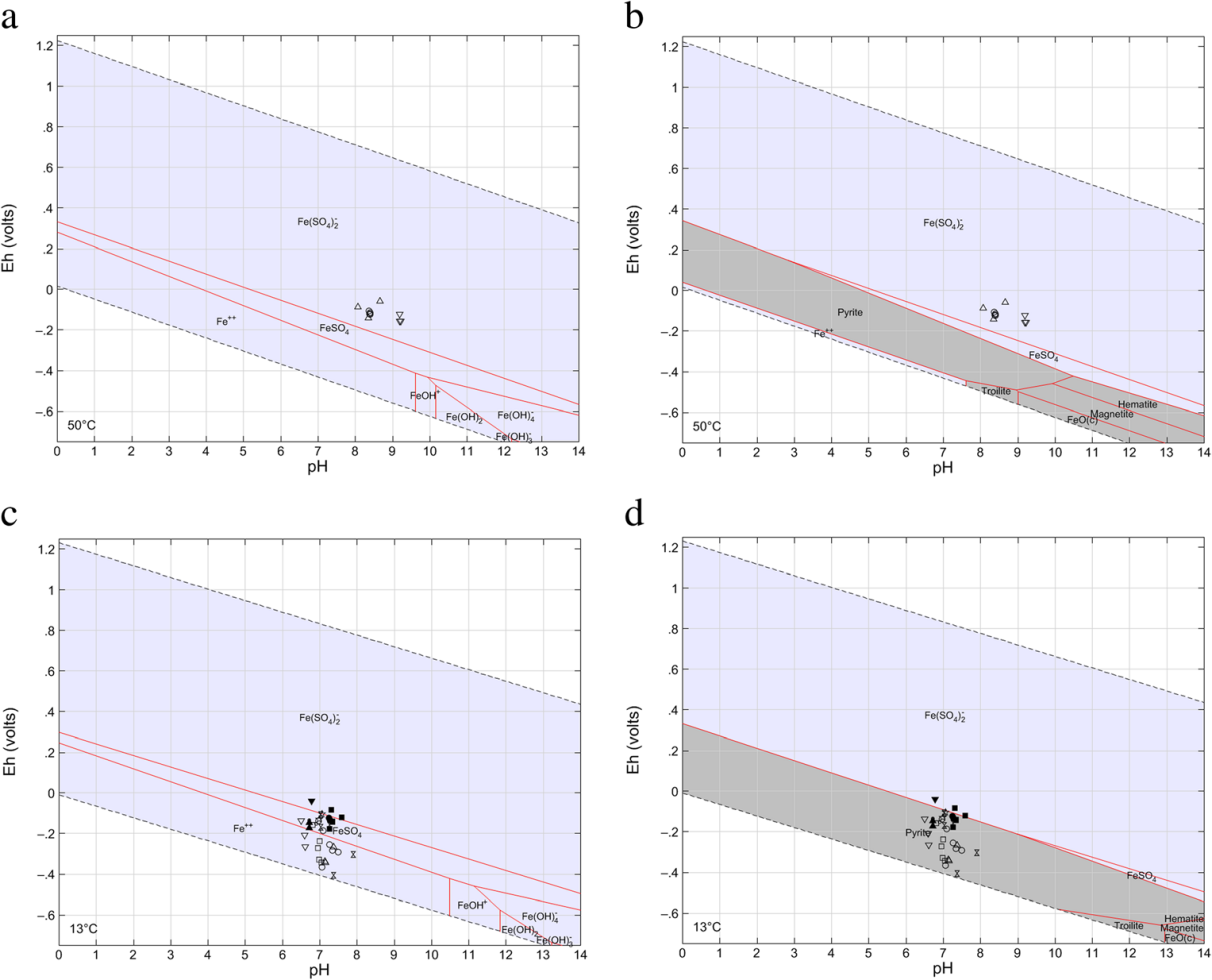
*E*_H_-pH diagrams of the iron species in groundwater at the Fe-S-O-H system. Thermal fresh waters (Sudetes Mountains), without water from C-1 intake (Cieplice), at 0.3 μM Fe, 1 mM S(VI), 10 μM S(II), 50 °C and 1 bar total pressure: **a** aqueous species and **b** aqueous and solid species. Mineral waters (Carpathian Foredeep), at 2 μM Fe, 20 mM S(VI), 1.5 mM S(II), 13 °C and 1 bar total pressure: **c** aqueous species and **d** aqueous and solid species. Filled symbols, waters of the Kraków region

In terms of iron species, all SM thermal waters (including C-1 well water in Cieplice) demonstrate Fe(SO_4_)_2_^2−^ as the predominant Fe aqueous species (Fig. 5a, b), whereas the chemistry of the CF mineral waters reveals Fe^2+^ and FeSO_4_^0^ as the predominant ones (Fig. 5c), wherein the latter form mainly in waters of the Kraków region (which have relatively higher ORP and lower S(II) concentrations than waters of the Busko region). All CF waters clearly express the stability of pyrite in the aquifer systems (Fig. 5d). The knowledge about the concentration of selected species of specific components is important because adequate speciation can affect the action of the therapeutic water. Researches indicate that the most effective in some treatments are mineral sulphide waters with the sulphur occurred in the form of hydrogen sulphide. This form presents a good pharmacological effect under low pH and low oxygen concentration conditions (Carbajo and Maraver 2017). Ferrous waters are more bioavailable when iron exists in a form of Fe^2+^ ions (Li et al. 2009; Ems and Huecker 2019). Iron-rich therapeutic waters (from Levico and Vetriolo spas, Trento, Italy) showed effectiveness and durability of therapy effects in the treatment of osteoarthritis and fibromyalgia (Cantarini et al. 2007; Fioravanti et al. 2013, 2018). Waters with high sulphates content have a good physiological action when SO_4_^2−^ is above 1200 mg L^−1^ (Gutenbrunner and Hildebrandt 1994; pp. 28, 177).

Various thermal waters of unchanged chemical composition and originally low ORP, containing increased sulphur or iron solutes, showed beneficial therapeutic effects, in contrast to the same waters affected by ageing (e.g. in open-air conditions) during water transfer and handling (Okouchi et al. 2010).

Groundwater differ each other in terms of many features of the occurrence environment, like depth of their presence, degree of isolation from the influence of surface conditions (i.e. recharging by precipitation water related to contemporary hydrological cycle, inflow of pollution), water flow velocity, time and rate of water turn-over in an aquifer system. The volume of water resources available for exploitation, water quality and vulnerability to possible contamination are closely associated with the abovementioned features. Part of therapeutic water resources might be regarded as a renewable one. This applies to groundwater in the active turn-over zone, i.e. in aquifers which are directly or indirectly recharged by water related to the modern hydrological cycle. Therapeutic waters, which occur in retarded water turn-over or stagnant water zones, are regarded as non-renewable. Nonetheless, one should be emphasized strongly that both types of therapeutic waters have a limited resource understood as the available flow rate (debit) or available total volume of water, respectively. This means the necessity to monitor their quantity and quality for proper managing and protecting the therapeutic water resources.

The exploitation of therapeutic waters always violates the natural physical and chemical status quo of the system and can lead to fluctuations and/or deterioration of water quantity and quality. Rationalizing the efforts and the costs of therapeutic water monitoring, the most informative, indicative and cheap-in-study parameters, like ORP, should be implemented and routinely tested. The ORP characterizes the resultant effects of all redox reactions, i.e. reactions in which electrons are transferred between aqueous (and solid) species, and it is one of few indicative parameters which quantitatively inform about the physicochemical status of the solution.

Chemistry of groundwater of the active turn-over zone commonly does not reach the full chemical equilibrium with aquifer rocks. Our results show that most of the studied waters reached equilibrium with aquifer rocks, which mainly results from their occurrence in a retarded water turn-over or stagnant water zones. Such kinds of waters present chemical status far from conditions in near-surface or surface environments. During groundwater exploitation (e.g. by pumping) and its further handling on the surface, inevitable changes in water quality might be expected, which reflect in ORP value.

Because the ORP results from and affects aqueous species of electro-active chemical elements, it helps understand the behaviour of the beneficial, balneologically appreciated components, like sulphur and iron solutes, in therapeutic waters. For the reasons summarized above, it should be recommended to monitor ORP in therapeutic water both at intake site and at treatment places, as it is for example required by Begriffsbestimmungen (2016).

Understanding oxidation-reduction conditions can be useful in various ways. For instance, a serious weakness of balneochemical research is too little attention paid to the presence and role of organic compounds in therapeutic waters (Varga 2011; Szabó and Varga 2019). In this field, ORP can provide important data about distribution and behaviour of organic species (e.g. Fekete et al. 2012). In the context of ORP studies, another example of an interesting proposition is assessing a reductive ability of therapeutic thermal waters by the volumetric method with povidone-iodine (Okouchi et al. 2011). This method provides complementary information, regarding the “classical” ORP measurement, and allows for estimating the chemical oxygen demand and the vitamin C equivalence with respect to the reductive ability of therapeutic waters.

## Conclusions

The ORP is one of the most important parameters characterizing hydrogeochemical conditions in the groundwater systems, like therapeutic water aquifers. ORP research provides information about the origin of therapeutic waters and might also be useful for the protection of these valuable natural resources. However, research on ORP in groundwater is rarely carried out, even in waters mandatory monitored, like therapeutic waters in health resorts. In core balneological literature, like Gutenbrunner and Hildebrandt (1994), the topic of ORP studying in therapeutic waters is not taken up.

The ORP is a parameter sensitive to measurement conditions and sometimes might give semi-quantitative information. Nevertheless, it is useful to make geochemical modelling based on the measured values or at least on theoretical ORP, since such calculations set the limits to the processes we may expect in natural and human-affected environments. The ORP measurements and geochemical modelling help explain the origin of therapeutic waters.

The occurrence forms of two important bio- and electro-active elements, sulphur(II) and iron, were evaluated in therapeutic waters basing on authors’ redox measurements and speciation-solubility geochemical modelling. Results indicate that in studied therapeutic waters presenting reducing conditions in the aquifers, sulphur and iron forms illustrate chemical equilibria achieved between groundwater and aquifer rocks. This might facilitate the identification of possible adverse physicochemical changes in the therapeutic water while handling to treatment places.

Characteristics of chemical conditions in exploited waters should be a starting point for the next assessments regarding changes in the therapeutic water quality when directing them to treatment places. The ORP should be monitored in therapeutic waters, for description and understanding the behaviour of both beneficial for human health and harmful elements in waters.

Summarizing, research on ORP in therapeutic waters provides important information in various aspects: (1) deciphering natural hydrogeochemical conditions in exploited aquifer, (2) evaluating possible physicochemical changes induced by exploitation, (3) tracking physicochemical changes in therapeutic water while transferring between a water intake and a treatment place and (4) evaluating physicochemical quality of waters applied in balneotherapy.
